# Genetic polymorphisms of bone marrow stromal cell antigen-1 (BST-1/CD157): implications for immune/inflammatory dysfunction in neuropsychiatric disorders

**DOI:** 10.3389/fimmu.2023.1197265

**Published:** 2023-05-29

**Authors:** Shigeru Yokoyama

**Affiliations:** ^1^ Research Center for Child Mental Development, Kanazawa University, Kanazawa, Japan; ^2^ Division of Socio-Cognitive-Neuroscience, United Graduate School of Child Development, Osaka University, Kanazawa University, Hamamatsu University School of Medicine, Chiba University and University of Fukui, Kanazawa, Japan

**Keywords:** anxiety, autism spectrum disorder, BST-1, CD157, neuroimmune dysfunction, Parkinson’s disease, single-nucleotide polymorphism

## Abstract

Bone marrow stromal cell antigen-1 (BST-1/CD157) is an immune/inflammatory regulator that functions as both nicotinamide adenine dinucleotide-metabolizing ectoenzyme and cell-surface signaling receptor. BST-1/CD157 is expressed not only in peripheral tissues, but in the central nervous system (CNS). Although its pathophysiological significance in the CNS is still unclear, clinical genetic studies over a decade have begun revealing relationships between BST-1/CD157 and neuropsychiatric diseases including Parkinson’s disease, autism spectrum disorders, sleep disorders, depressive disorders and restless leg syndrome. This review summarizes the accumulating evidence for the involvement of BST-1/CD157 in these disorders.

## Introduction

Bone marrow stromal antigen-1 (BST-1/CD157) is a cell-surface membrane molecule that promotes pre-B lymphocyte growth (1, 2). BST-1/CD157, along with its paralogue CD38, constitutes a nicotinamide adenine dinucleotidase (NADase)/ADP-ribosyl cyclase family (2–10). These two enzymes catalyze the synthesis of cyclic ADP-ribose (cADPR) from NAD^+^ and thereby regulate the intracellular Ca^2+^ homeostasis (8–10). Also, BST-1/CD157 has a base-exchange activity for nicotinamide riboside and nicotinic acid riboside (11). In addition to these enzymatic activities, BST-1/CD157 as well as CD38 serves as a cell-membrane receptor that transmits signals for cell polarization, migration, and diapedesis (12).

BST-1/CD157 is expressed by myeloid lineage cells including neutrophils, eosinophils, basophils and macrophages in the peripheral blood, and by B-cell and myeloid precursors in the bone marrow (2, 10, 12–17). Its expression has also been reported in other tissues, such as peripheral mesothelium (18), vascular endothelium (19, 20) and Peyer’s patches (21). BST-1/CD157 thus plays diverse roles in humoral immune responses, leukocyte transmigration, and the maintenance of hematopoietic, intestinal and vascular endothelial stem cells (2, 12–21).

More importantly, BST-1/CD157 holds much pathogenetic and clinical significance in various diseases including autoimmune diseases, hematologic malignancies and solid tumors (10, 17). Nurse-like cells cloned from bone marrow and synovial tissues of patients with rheumatoid arthritis promoted survival of peripheral B cells, which was significantly blocked by anti-BST-1/CD157 antibody; and recombinant soluble BST-1/CD157 showed a similar survival effect (2, 22). It has been also demonstrated that BST-1/CD157 is involved in the progression and differentiation of leukemia (23–25), metastasis of ovarian carcinoma cells (26–28), malignant mesothelioma (29, 30) and glioma (31), and thus could be used as diagnostic or prognostic markers. Particularly, BST-1/CD157 has been regarded as a target for immunotherapy of acute myeloid leukemia (23–25). Despite the advances in the study of these diseases, it remains unclear whether BST-1/CD157 is involved in the pathogenesis of neuropsychiatric disorders in humans.

In this review, I survey the past studies on the *BST-1/CD157* gene and discuss over its implications in neuropsychiatric disorders.

## Structure of the human *BST-1/CD157* gene and its expression in the nervous system

**Figure 1:**
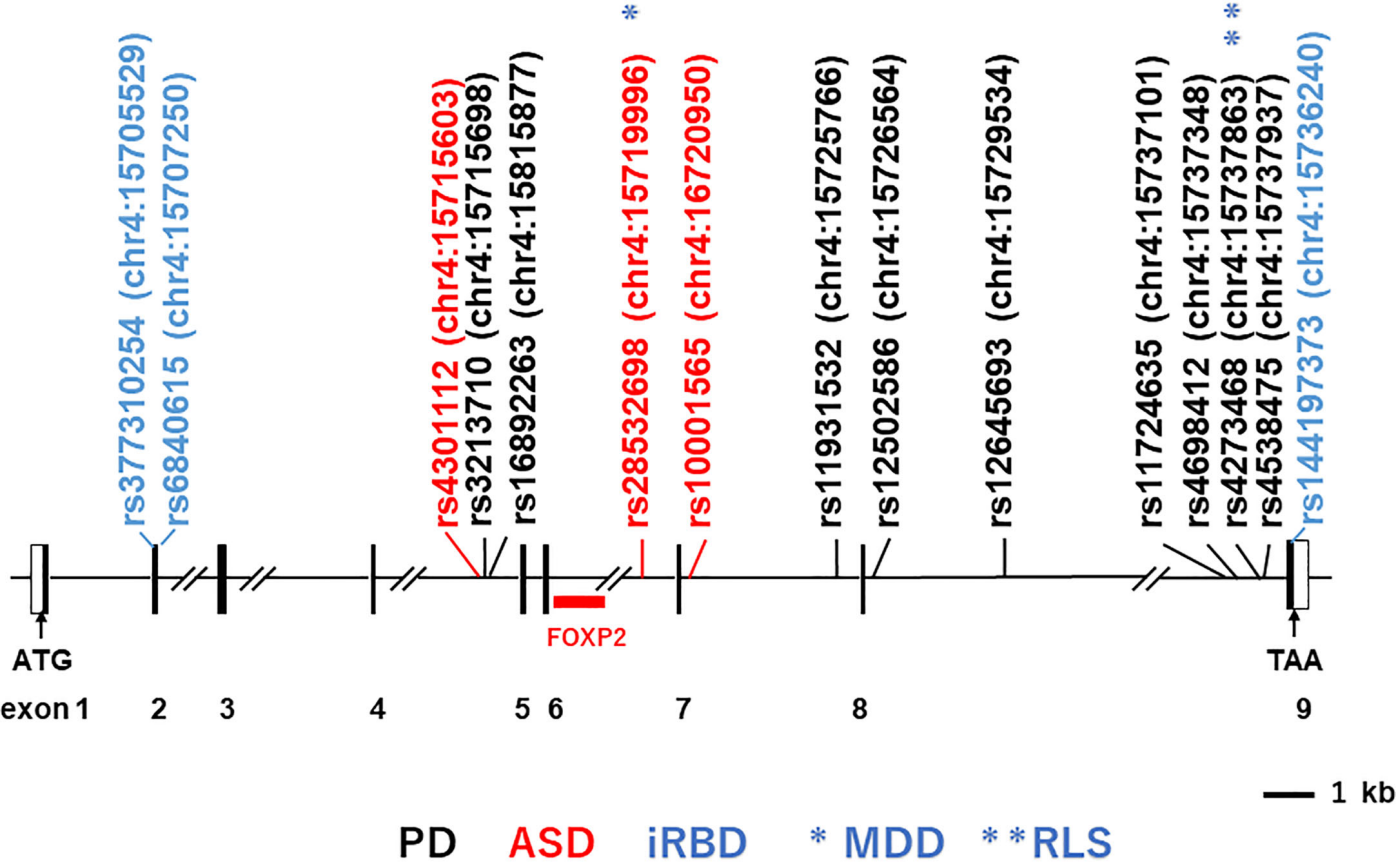
Structure of the human *BST-1/CD157* gene and locations of main single-nucleotide-polymorphisms (SNPs). Depicted is the exon-intron organization based on GenBank accession numbers NM_004334 and NC_000004. Black and open boxes represent protein-coding regions and untranslated regions, respectively. The locations of the SNPs on human chromosome 4 (chr4) are indicated *in parentheses*; numbers after colons represent genomic positions based on the human genome assembly the UCSC GRCh38/hg38 genome browser (http://www.genome.ucsc.edu/cgi-bin/hgGateway?db=hg38). SNPs *in black, red* and *blue* stand for those reported to be associated with Parkinson’s disease (*PD*; representative ones), autism spectrum disorder (*ASD*) and isolated REM sleep behavior disorder (*iRBD*), respectively. Single asterisk and double asterisks (*in blue*) represent association with major depressive disorder (*MDD*) and restless leg syndrome (*RLS*), respectively.

The human *BST-1/CD157* gene maps to the short arm of chromosome 4 (4p15.32), where its paralogue *CD38* gene is also located. The major transcript for BST-1/CD157 is encoded by nine exons that encompass over 35 kb in this chromosomal region (**Figure 1**).

Although BST-1/CD157 exists widely in both lymphoid and non-lymphoid tissues including blood, bone marrow, thymus, spleen, lymph nodes, lung, liver, gut, uterus, and vascular endothelial cells (8, 10, 17), little is known about its expression in the nervous system. RNA blot hybridization analysis in earlier studies did not detect BST-1/CD157 mRNA in human and mouse brains (1, 3). According to the Human Protein Atlas (32, 33), BST-1/CD157 mRNA is detectable in the normal human brain at low levels without regional specificity. Our immunohistochemical staining detected BST-1/CD157-immuoreactivity in the amygdala and somatosensory cortex of mice (34, 35). To date, changes in BST-1/CD157 expression in inflamed CNS have not fully been examined.

## Parkinson’s disease

Parkinson’s disease (PD) is a common and complex neurological disorder that exhibits classical motor dysfunctions, including bradykinesia, resting tremor and gait disturbance, and non-motor features, such as psychiatric symptoms, sleep disorder and cognitive impairment (36). Epidemiological studies have revealed that both genetic and environmental factors are attributable to PD (36–41).

The initial genome-wide association study (GWAS) in a Japanese population reported rs11931532, rs12645693, rs4698412 and rs4538475 in the *BST-1/CD157* gene as risk SNPs for sporadic late-onset PD (**Figure 1**; **Table 1**) (44). Afterwards, studies in various ethnicities have identified nearly ten PD-associated SNPs (**Table 1**). Among them, two SNPs, rs11724635 and rs4698412 (**Figure 1**; **Table 1**), have been examined most repeatedly. The statistically significant association of rs1573458 has been observed in six subsequent studies (**Figure 1**; **Table 1**) (51–57), but not in Asian and Caucasian cohorts (45, 47, 58–60).

**Table 1:** Parkinson’s disease-associated SNPs tested in the *BST-1/CD157* gene.

SNP^a^	Position^b^	Region	Association^c^	Countries/Ethnicity	References
rs3213710	15715698	Intron 4	Yes	Canada, France, USA, Israel	(42)
rs16892263	15715877	Intron 4	Yes	Korean	(43)
rs11931532	15724143	Intron 7	Yes	Japan Asian	(44) (45)
			No	Chinese Japan	(46) (47)
rs12502586	15724941	Intron 8	Yes	Netherlands Ashkenazi Jewish	(48) (49)
			No	European origin	(50)
rs12645693	15727911	Intron 8	Yes	Japan Ashkenazi Jewish	(44) (49)
			No	Japan	(47)
rs9790670	15731374	Intron 8	Yes	Korean	(43)
rs11724635	15735478	Intron 8	Yes	Meta-analysis (USA, Germany, UK and France) Caucasian Asian, Caucasian China Combined (USA, Irish and Polish) European origin USA, Canada	(51) (52) (53) (54)^d^ (55) (56) (57)
			No	Japan Taiwanese Chinese meta-analysis (Asian, Caucasian) Asian	(47) (58) (59) (60) (45)
rs4698412	15735725	Intron 8	Yes	Japan USA (European origin) China Ashkenazi Jewish European UK White, non-Hispanic Chinese meta-analysis (Asian, Caucasian) Asian China China	(44) (61) (62) (49) (63) (64) (65) (66) (60) (45) (67) (68) (45)
			No	European origin Chinese	(50) (46)
rs4273468	15736240	Intron 8	Yes	Chinese Chinese	(69) (70)
rs4538475	15736314	Intron 8	Yes	Japan Ashkenazi Jewish Asian	(44) (49) (52)
			No	Chinese	(71)
rs2302468	15703251	Exon 1, p.G36A	No	Chinese	(72)
rs78449217	15707565	Exon 3, p.R124C	No		
rs2302465	15707569	Exon 3, p.R124H	No		
rs2302464	15707629	Exon 3, p.145Q	No		
rs2302463	15711823	Exon 4, p.S156S	No		
rs1058212	15731831	Exon 9, p.R315R	No		
rs4698120	15743332	Downstream	Yes	Korean	(43)

a IDs are from dbSNP of the National Center for Biotechnology Information.

b Position on the chromosome is based on the GRCh38 (GCF_000001405.26).

c Association represents statistical significance in a case-control study.

d Significant association was observed only in minor allele frequency.

The association of rs4698412 has been confirmed in eleven subsequent studies in populations with different ethnic backgrounds (45, 49, 60–68), but was not in European (50) and Chinese cohorts (46).

In search of PD-associated SNPs in exons, Wang et al. re-sequenced all the 9 exons of the *BST-1/CD157* gene in a Chinese cohort. Of 524 PD cases and 527 controls, 6 non-synonymous SNPs were identified in exons 1, 3, 4, 7, and 9; but their association was insignificant (72). Thus, all PD-associated SNPs identified so far are located in introns, making it difficult to define a causal relationship between these SNPs and the pathogenesis of PD. In addition, all the SNPs in this review represent common variation in normal population, with their minor allele frequency being more than 10%. Hence any of them alone could not be an appropriate diagnostic or prognostic biomarker for PD. It is worth examining, however, whether these SNPs could be integrated effectively into polygenic risk score analysis (73) in combination with SNPs of *IL-6, TNF-α* and many other PD-related genes (41).

## Autism spectrum disorder and other diseases

Autism spectrum disorder (ASD) is a neurodevelopmental disorder characterized by social communication deficits and restricted repetitive behaviors with a strong genetic inheritability as well as other environmental causes (74–76). An initial notable report was on a patient with both autistic symptoms and asthma (77). In this case, an 84-kb deletion between the *BST-1/CD157* and *CD38* genes resulted in an in-frame *BST-1/CD157* and *CD38* fusion transcript (77). One hypothetical explanation is that disruption of the *CD38* gene in the vicinity reduced cyclic ADP-ribose formation, resulting in dysfunctional calcium (Ca^2+^)-induced Ca^2+^-release for the secretion of oxytocin, a neurohypophyseal hormone for social behavior and recognition (78–80); however, the functional consequence of this fusion transcript is unknown.

We subsequently reported association between 3 SNPs (rs4301112, rs28532698, and rs10001565) located in the *BST-1/CD157* gene with ASD (**Figure 1**; **Table 2**) (81). This case-control study in a Japanese population tested genetic association between 93 SNPs in the *BST-1/CD157* gene and ASD, and found out these possible risk SNPs. These SNPs are located separately from Parkinson’s disease-associated ones. As they are in high linkage disequilibrium (81), it is likely that the results represent single underlying pathogenetic process.

**Table 2:** *BST-1/CD157* gene SNPs tested in other neuropsychiatric disorders.

SNP^a^	Position^b^	Region	Association^c^	Countries/Ethnicity	References
Autism spectrum disorder						
rs4301112	15715603	Intron 4	Yes	Japan	(81)	
			No	Chinese	(82)	
rs28532698	15719996	Intron 6	Yes	Japan meta-analysis	(81) (83)	
			No	Chinese	(82)	
rs10001565	15720950	Intron 7	Yes	Japan	(81)	
REM sleep behavior disorder						
rs377310254	15705579	Exon 2, p.V85M	Yes	European	(84)	
rs6840615	15707250	Exon 2, p.I101V	Yes			
rs144197373	15736240	Exon 8, p.V272M	Yes			
Major depressive disorder						
rs28532698	15719996	Intron 6	Yes	Taiwan	(85)	
Restless leg syndrome/Willis-Ekborn disease						
rs4273468	15736240	Intron 8	Yes	Chinese	(86)	
Alzheimer’s disease (sporadic, late-onset)						
rs11724635	15735478	Intron 8	No	Chinese	(87)	

a IDs are from dbSNP of the National Center for Biotechnology Information.

b Position on the chromosome is based on the GRCh38 (GCF_000001405.26).

c Association represents statistical significance in a case-control study.

Bioinformatic analysis of the *BST-1/CD157* gene using the HaploReg program (88, 89) predicts that genetic variations at these three SNPs may be associated with altered binding of neural development-related transcription factors: histone deacetylase C2 (HDAC2) (90), POU class 6 homeobox 1 (POU6F1) (91), and hes-related family bHLH transcription factor with YRPW motif 1 (HEY1s) (92), respectively. In addition, in the UCSC (GRCh37/hg19) track “Transcription Factor ChIP-seq (161 factors) from ENCODE (93) with Factorbook Motifs”, the region between rs4301112 and rs10001565 [chr4:15717226–15722573 (corresponding to chr4:15715603–15720950 in GRCh38/hg38)] includes potential binding sites for c-Jun, STAT3 (signal transducer and activator of transcription 3), FOXP2 (forkhead box protein P2) (**Figure 1**), PolR2a (Polκ RNA polymerase II polypeptide A), Elf-1 (E74-like factor 1), HNF4G (hepatocyte nuclear factor 4 gamma), HNF4A (hepatocyte nuclear factor 4 alpha), JunD, and C/EBPβ (CCAAT/enhancer binding protein beta). These potential regulatory sites are overlapped with a peak of H3K27Ac Mark track, where acetylation of lysine 27 of the H3 histone protein is assumed to regulate brain development at the level of transcription (94, 95). In particular, FOXP2 seems important because its genetic abnormalities have been implicated in speech and language disorders (96, 97). A chromosomal translocation disrupting the FOXP2 gene and an amino-acid substitution in its forkhead domain have been demonstrated in patients with severe developmental disorders of speech and language (96). FOXP2 mRNA is expressed in the developing human brain, in good concordance with anomalous sites identified by brain imaging in adult speech and language disorders (97). It is thus tempting to postulate that BST-1/CD157 expression is mediated by FOXP2 during the early brain development.

**Figure 2:**
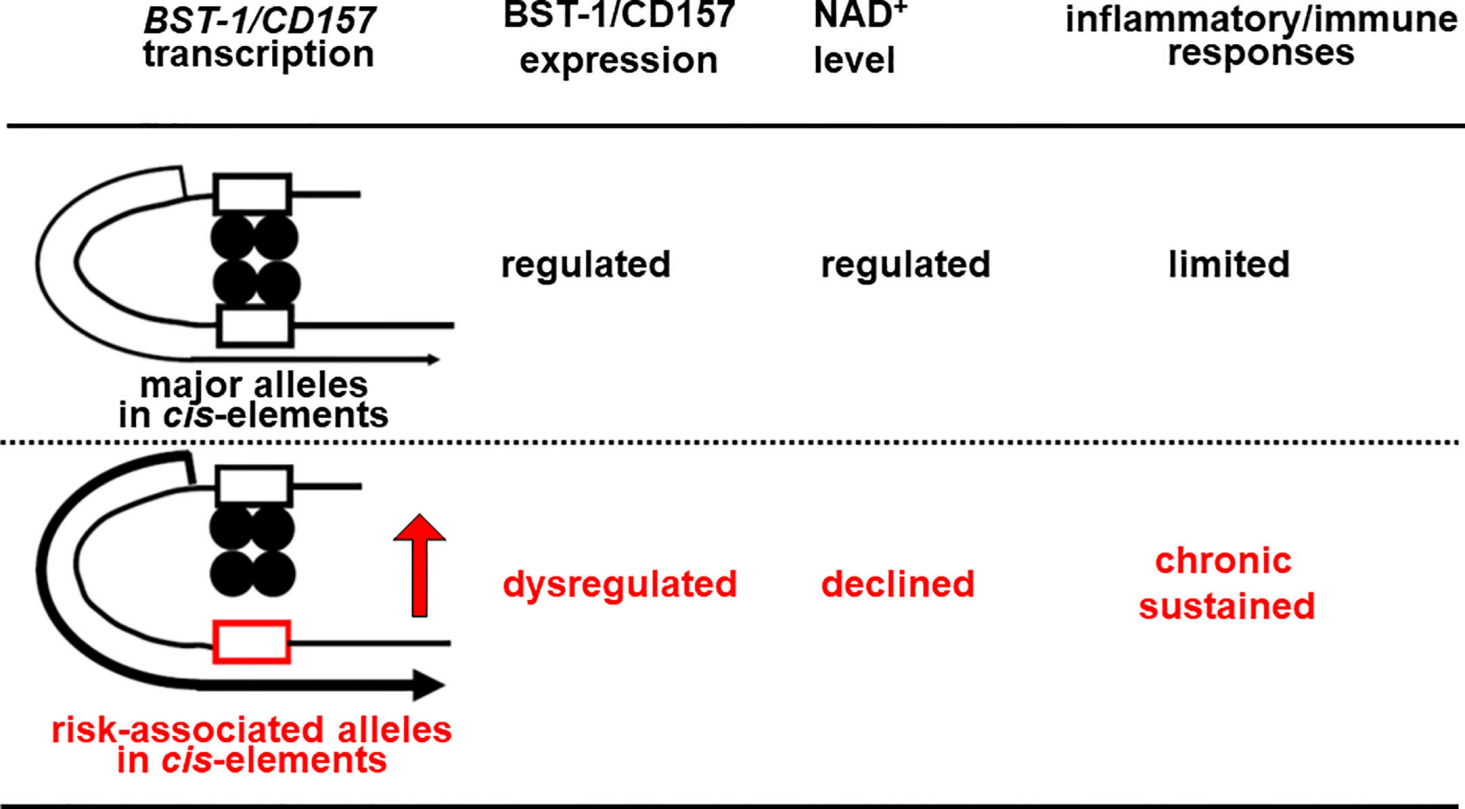
Hypothetical scheme for Bst-1/CD157-mediated inflammatory/immune regulation in the CNS. Nucleotide substitution(s) may lower binding affinities of transcription factors (*closed circles*) to in *cis*-regulatory regions (*open boxes*), decrease repressive effects on transcription and thereby upregulate the expression of the *BST-1/CD157* gene, presumably in myeloid cells migrated from the periphery and/or microglia. This would disrupt the NAD^+^ homeostasis in the CNS, resulting in sustained immune/inflammatory reaction.

In other genes, these factors as well as FOXP2 are known to repress transcription through binding to *cis*-regulatory elements (90–92, 94, 95, 97). Currently, however, there is no data for their binding to *cis*-regulatory elements in the *BST-1/CD157* gene. Also, it remains unknown whether genetic variation(s) in the *BST-1/CD157* gene can change their repressive effect. I would hypothesize that nucleotide substitution(s) reduce binding affinities, weaker repressive effects on transcription and thereby dysregulate (possibly upregulate) expression of *BST-1/CD157*. As in the increase in CD38 and decrease in NAD^+^ (98–100), disruption of the NAD^+^ homeostasis would result in sustained immune/inflammatory reactions (**Figure 2**).

It is now well known that sustained immune/inflammatory activation is observed in the brain of the patients with developmental disorders and neurodegenerative diseases (101). Vargas et al. reported activation of microglia and astrocytes in autistic patients (102). Thus, it would be worth examining whether BST-1/CD157 is involved in such pathological state.

Interestingly, the ASD-associated SNP rs28532698 also showed association with major depressive disorder (MDD) in a Taiwan population (**Figure 1**; **Table 2**) (85). Huang et al. found that rs4273468 increased the risk of idiopathic restless leg syndrome (RLS)/Willis-Ekbom disease (WED) patients in a southeastern Chinese population (86). Although rs4273468 is also associated with PD (**Figure 1**; **Table 2**), relationship between this common sleep related movement disorder and PD remains unknown (103). Also, Mufti et al. reported that rare coding SNPs in the *BST-1/CD157* gene, together with rare noncoding variants in the *LAMP3* (lysosomal associated membrane protein 3) gene, was associated with isolated REM sleep behavior disorder (iRBD; **Table 2**) (84). All these non-synonymous variants (p.V85M, p.I101V, and p.V272M) seem to be loss-of-function variants with a potential effect on the protein structure and stability.

## Shared genetic architecture and phenotypic traits

As above, SNPs in the *BST-1/CD157* gene have been reported to be associated with at least five different neuropsychiatric diseases: Parkinson’s disease, ASD, iBRD, MDD and RLS. This multiple association could be regarded as genetic pleiotropy in which one genetic variant has influence on more than one phenotype (104, 105). Although both common and rare genetic variants are known to show genetic pleiotropy, this phenomenon is more frequently demonstrated in common variants than in rare variants (105). In consistent, with the exception of the exonic SNPs in iBRD, most risk alleles are common ones with frequencies > 1% in general human populations.

In the current conception, many common variants, each of which has a small effect size, in sum could be genetic risk of psychiatric neuropsychiatric disorders; in contrast, rare variants possess a large effect size, and one or small number of such variants are sufficient to cause disorders (104, 106). In most case-control studies of *BST-1/CD157* SNPs, odds ratios have been estimated less than 2, suggesting that the *BST-1/CD157* variations identified so far have a small effect size in the pathogenesis of common polygenic neuropsychiatry disorders.

The most common phenotypic trait among the five disorders is anxiety. In mice deficient in the *BST-1/CD157* gene, Lopatina et al. reported anxiety-related and depression-like behaviors without apparent motor dysfunction, along with communication impairment (34, 35, 107, 108). These behaviors were alleviated by the treatment with anxiolytic agents, such as benzodiazepines (109), monoamine oxidase B inhibitors (109) and oxytocin (34, 107, 110). CD157 was weakly expressed in the amygdala and c-Fos-immunoreactivity, an indirect marker of neuronal excitability, which was less evident in BST-1/CD157-knockout (*BST-1/CD157 -/-*) mice than in wild-type mice (34). These observations in mice suggest that altered BST-1/CD157 expression in a certain brain region might affect mental state.

## Conclusion and perspectives

In the past decade, an increasing number of genetic studies have suggested that the *BST-1/CD157* gene could be a risk locus for several different neuropsychiatric disorders including PD and ASD. Future studies should define the nature of shared influences of BST-1/CD157 between psychiatric disorders and other diseases and phenotypic traits, especially immune/inflammatory dysfunction. The existing data, however, indicate nothing more than correlation between genetic variation and diagnoses. While the role of BST-1/CD157 variation in the genetic architecture of neuropsychiatric diseases has become clearer, the underlying molecular mechanisms remain elusive. At the same time, the physiological functions of BST-1/CD157 in the brain are still unclear. It is necessary to analyze BST-1/CD157 expression and their regulatory processes in the both developing and inflamed brain in detail.

Moreover, influences of BST-1/CD157 in the periphery on the CNS should be explored more extensively. A flurry of recent reports has documented microbiome-gut-brain axis (111, 112). Changes in gut microbiota has been shown to modulate anxiety (113, 114), depression (113, 114) and core symptoms of ASD (115, 116). Given its regulatory roles in the immune/inflammatory reactions (2, 12, 13, 117) and in the renewal of intestinal stem cells (21), it is conceivable that altered BST-1/CD157 activity may dysregulate conditions of the gut and enteric nervous system and thus result in mental disorders.

## Author contributions

SY conceived, wrote and revised the manuscript. The author confirms being the sole contributor of this review article and has approved it for publication.
